# A Modified Single Mini-Incision Complete Urinary Tract Exenteration for Urothelial Carcinoma in Dialysis Patients

**DOI:** 10.1155/2014/649642

**Published:** 2014-08-11

**Authors:** I-Hsuan Chen, Jen-Tai Lin, Jeng-Yu Tsai, Tony Wu, Chia-Cheng Yu

**Affiliations:** ^1^Division of Urology, Department of Surgery, Kaohsiung Veterans General Hospital, 386 Ta-Chung 1st Road, Kaohsiung 813, Taiwan; ^2^Division of Urology, Department of Surgery, Tri-Service General Hospital, National Defense Medical Center, Taipei 114, Taiwan; ^3^School of Medicine, National Yang-Ming University, Taipei 112, Taiwan; ^4^Department of Pharmacy, Tajen University, Pingtung 90741, Taiwan

## Abstract

*Objective*. To present our experience with single mini-incision complete urinary tract exenteration (CUTE) for female dialysis patients suffering from urothelial carcinoma (UC). *Patients and Methods*. Institutional review board approval was obtained. From 2005 through 2012, 14 female dialysis patients with UC underwent single mini-incision CUTE, in combination with radical hysterectomy and bilateral salpingo-oophorectomy. All were placed in the modified dorsal lithotomy position without repositioning. An infraumbilical midline mini-incision was made. Bilateral nephroureterectomy was first performed entirely extraperitoneally, followed by radical cystectomy with removal of the uterus and ovaries transperitoneally. *Results*. All procedures were done successfully without major complications. The median operative time was 242.5 minutes, and estimated blood loss was 500 mL. The median time to oral intake was 2 postoperative days; the median hospital stay was 11 days. Ten patients remained cancer-free at a median follow-up of 46.5 months; six patients were confirmed as having preoperatively undetectable UC or renal cell carcinoma, even after reviewing preoperative computed tomography. *Conclusions*. This modified technique provides a time-saving complete urinary tract extirpation to eliminate preoperatively undetectable malignancy, reduce metachronous recurrences, and avert perioperative complications associated with pneumoperitoneum and repositioning. Good cancer control and early convalescence can mutually be achieved in experienced hands.

## 1. Introduction

In Taiwan, there is an increased risk for urothelial carcinoma (UC) in patients of end-stage renal disease (ESRD), with the incidence ranging from 0.89% to 2.1%, especially women aged 50 years or younger [1]. On account of its high recurrence rate and rapidly progressive behavior among dialysis patients, total urinary tract exenteration is a recommended treatment modality to reduce the incidence of metachronous multicentric UC [2]. Besides, lack of suitable imaging studies for follow-up of the upper tract and the probability of morbidity related to stepwise urinary tract extirpation support the more aggressive surgical strategy for treating high-risk patients with ESRD.

Conventionally, complete urinary tract exenteration (CUTE) is performed via a long midline incision extending from the xiphoid process to the pubic symphysis or a bilateral flank approach followed by a midline infraumbilical laparotomy incision. Long operative time and high surgical risks were of great concern at that time. With the advent of laparoscopic techniques and the experience of open surgery, we develop a single mini-incision unilateral nephroureterectomy with bladder cuff excision, via an infraumbilical midline incision, for upper tract UC. By applying this method to synchronous bilateral nephroureterectomy, CUTE, through a single mini-incision approach, in single session can be the treatment option of choice as if there are indications for radical cystectomy in female patients receiving dialysis. Removal of gynecological organs may be undertaken simultaneously in peri- and postmenopausal patients.

In the present study we describe our modified technique in treating female dialysis patients with urothelial carcinoma. Throughout the whole course, patient repositioning or rotating the operating table with cuff inflation is not needed; the specimens were extracted* en bloc* from the infraumbilical midline incision. We also compare our results with those of similar studies.

## 2. Patients and Methods

From 2005 through 2012, a total of 14 female dialysis patients underwent single mini-incision CUTE, radical hysterectomy, and bilateral salpingo-oophorectomy at our institution. Ten of them had multifocal UC (9 synchronous upper tract and bladder, 1 bilateral upper tract); 3 patients had organ-confined bladder UC (clinical stage T1, T2, or carcinoma in situ) with left-sided hydronephrosis; the remaining one only had urinary bladder UC. No lymph node or distant metastasis was noted preoperatively.

### 2.1. Extraperitoneal Preparation

**Figure 1:**
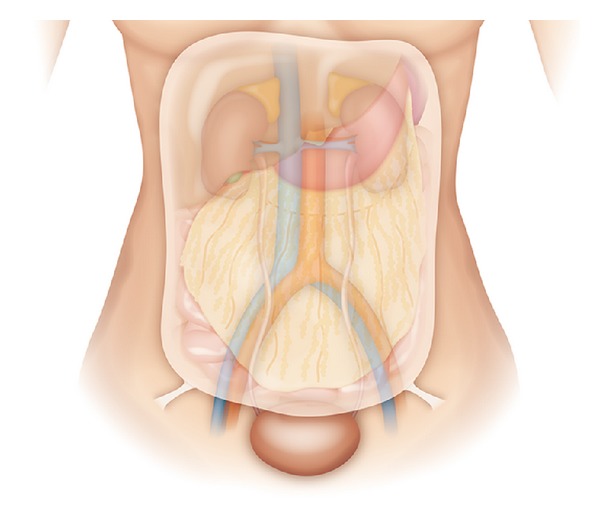
Illustration of two cord-like structures passing forwards in the peritoneal fold and entering the inguinal canal by the internal ring, considered to be the round ligament or spermatic cord in the female and the male, respectively.

**Figure 2:**
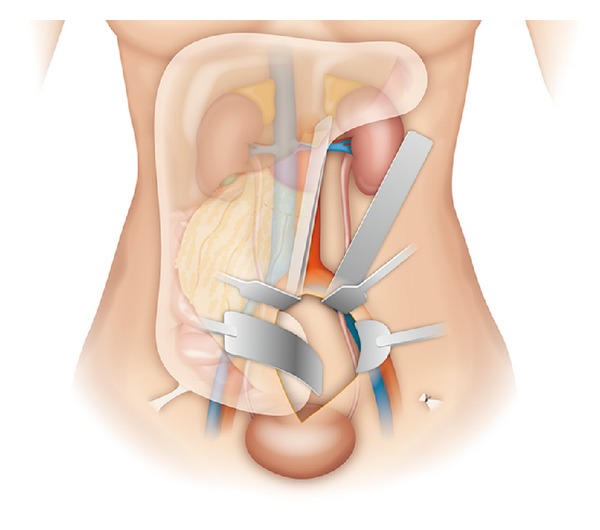
Illustration of* Peritoneal mobilization*. After division of the round ligament, two customized long right-angle retractors (upper 2 blades), as well as a Bookwalter retractor system (lower 2 blades), are used to push the peritoneum medially and anteriorly, thereby exposing the retroperitoneum.

All patients were placed in the modified dorsal lithotomy position with both legs supported in stirrups. An infraumbilical midline incision, about 10 cm in length, was made. The posterior rectus fascia was carefully dissected to gain access to the extraperitoneal space, which was then created from the pubic symphysis cephalad using blunt dissection and sweeping method. Once adequate working space was obtained with visualization of the psoas muscle, bilateral round ligaments attached to the peritoneum were identified, ligated, and divided (Figure 1). The retroperitoneal space could be widely explored using a Bookwalter retractor system (Codman, USA), in company with two customized long right-angle retractors (blade 15 or 23 cm). One was used to lift the abdominal wall up and the other sweeping the peritoneum and its contents medially (Figure 2).

### 2.2. Bilateral Nephroureterectomy

**Figure 3:**
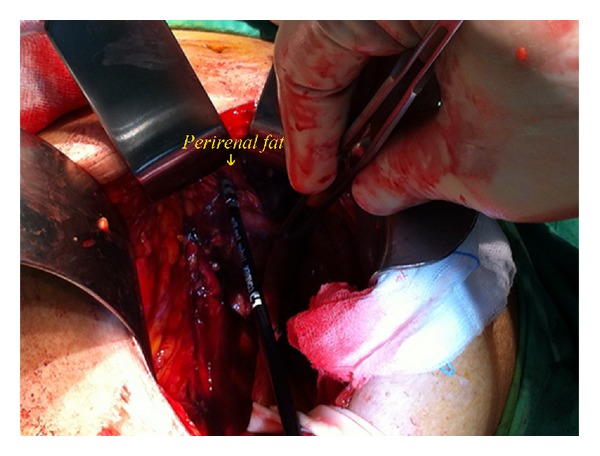
Intraoperative image of* Perirenal dissection*. A circumferential dissection of the kidney was performed along the plane between the renal capsule and the perinephric fat, with assistance of LigaSure.

**Figure 4:**
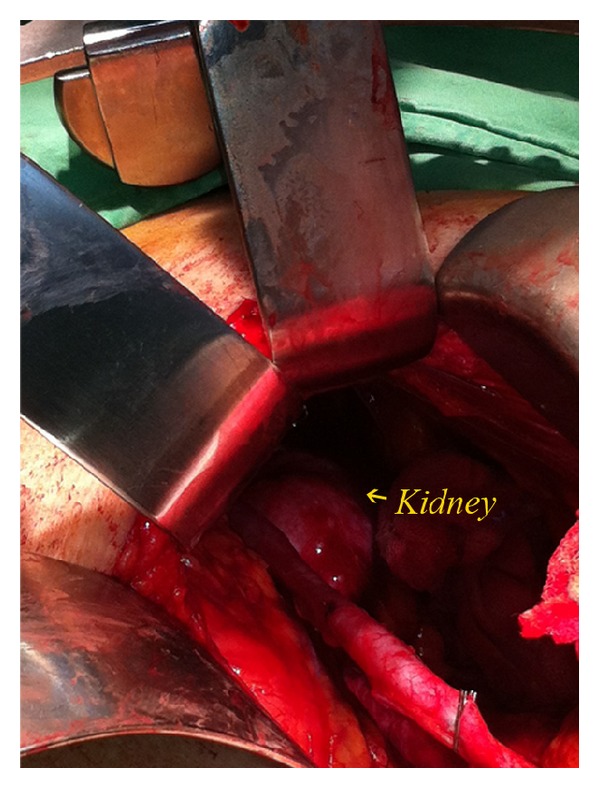
Intraoperative image showing good exposure of the retroperitoneum and complete mobilization of the kidney.

The ureter on one side was first identified, and ureteral skeletonization was performed cephalad using electrocautery toward the renal pelvis. After exploring the lower pole of the ipsilateral kidney, a circumferential dissection was performed manually along the plane between the renal capsule and the perinephric fat (Figure 3). Besides, bipolar electrocautery would be used to facilitate dissecting cephalad from the lower pole of the kidney toward the upper pole, by dividing any side-wall attachments. As the kidney was completely mobilized (Figure 4), the renal pedicle was isolated, ligated, and divided* en bloc* with Endo-GIA staplers (US Surgical Corporation, Norwalk, Connecticut), and the kidney was retrieved through the midline incision. The same procedure was repeated on the other side.

### 2.3. Radical Cystectomy

Radical cystectomy for women traditionally includes removal of the uterus, bilateral fallopian tubes, ovaries, and part of the vagina. After bilateral extraperitoneal nephroureterectomy, transperitoneal cystectomy, radical hysterectomy, bilateral salpingo-oophorectomy, and pelvic lymph node dissection were accomplished using standard open surgical techniques. Frozen section pathology of the bladder neck was performed to ensure a safe margin. The entire specimens were* en bloc* retrieved from the infraumbilical midline wound, and the urothelial continuity was maintained intact.

All surgeries were executed by one single surgeon (C. C. Yu). Follow-up abdominal computerized tomography (CT) was performed 3 months after surgery, every 6 months for the next 3 years, and then annually for life. With approval of the institutional review board, patient demographics and perioperative parameters, including operative time, blood loss, and convalescence and cancer control, were retrospectively reviewed and compared with peer-reviewed literature. Continuous variables were compared with the one sample *t*-test and categorical variables using the chi-square test. *P* < 0.05 was considered statistically significant.

## 3. Results

Patient demographics and perioperative outcomes were shown in Table 1. All patients were in good performance status (0-1), and all procedures were done successfully without major complications. The median duration of dialysis was 8.5 (6, 10.75) years. Ten patients remained cancer-free at a median follow-up of 46.5 (30.25, 87) months; four patients died of nonmalignant causes. Among ten patients with multifocal urothelial carcinoma, three (number 5, 8, 9) had incidental UC in the upper tract and one (number 1) was diagnosed as having a 1 mm Furhman grade I clear cell renal cell carcinoma (ccRCC) at the left kidney. Muscle-invasive bladder cancer with concomitant bilateral ureteral UC was incidentally found in one patient (number 11) presenting with recurrent bladder tumors and left hydronephrosis. Concurrent unilateral upper tract UC was also incidentally noted in the patient (number 13), who underwent CUTE for primary bladder cancer. All these preoperatively undetectable tumors were confirmed after carefully reviewing preoperative CT scans and postoperative histopathology. The median operative time was 242.5 (187.5, 268.75) minutes, and estimated blood loss was 500 (325, 750) mL. The median time to oral intake was 2 (1.75, 2) postoperative days, and the median hospital stay was 11 (9, 13.5) days. Postoperative complications included two cases of postoperatively prolonged ileus and one esophageal ulcer. No arteriovenous fistula formation was noted on follow-up CT scans after* en bloc* ligation of the renal pedicle. Statistical comparison of variables between different studies was shown in Table 2 [3–9]. In comparison with similar operations [7–9], our operative time and the interval to oral intake were significantly shorter; blood loss, hospital stay, and complications were insignificantly different. Except for more blood loss, our results were comparable to those of other smaller-scale surgeries in terms of operative time and convalescence [3–6].

## 4. Discussion

Urothelial carcinoma (UC) is the most common malignancy in dialysis patients of Taiwan [10]. On account of its high recurrence rate and rapidly progressive behavior, a more aggressive surgical strategy is recommended to improve the quality of life and prolong the survival of these patients [2, 11]. Complete urinary tract exenteration (CUTE) in single session is recommended for avoiding multistaged surgeries, associated with repeated analgesia, intraabdominal adhesions, delay in treatment, and higher morbidities and mortalities. Bothersome results, such as positive urine cytology and a suspicious filling defect within the urinary tract, and unpleasant follow-up procedures, like cystoscopy and retrograde pyelography, can be precluded and possible complications related to a contracted urinary bladder and nonfunctioning kidneys may be prevented as well. Traditional CUTE was performed through a long transperitoneal midline incision extending from the xiphoid process to the pubic symphysis. With the improvement of laparoscopic techniques and instrumentation, minimally invasive therapies may be offered [7–9].

Berglund et al. [12] first published the feasibility of laparoscopic radical cystoprostatectomy and bilateral nephroureterectomy for 2 male patients in 2005. Thereafter, Ou and Yang [7], Li et al. [8], and Lin et al. [9] successfully accomplished transperitoneoscopic or retroperitoneoscopic CUTE for dialysis patients. Pneumoperitoneum is the essential component for laparoscopy, but potential risks related to hypercapnia, cardiopulmonary compromise, hypothermia, subcutaneous emphysema, and air embolism exist. Besides, uremic patients on chronic dialysis often present with multiple comorbidities, including anemia, diabetes mellitus, cardiovascular disease, peptic ulcer disease and platelet dysfunction, and increasing perioperative morbidity and mortality. The extent of the surgery such as CUTE represents a considerable challenge to the patient, surgeon, and anesthesiologist. In order to reduce hemodynamic fluctuations related to this major high-risk surgery, pneumoperitoneum was replaced by a modified retractor system, and no 90-day postoperative mortality was reported in our series.

Tracing back to our history of evolution, unilateral hand-assisted retroperitoneoscopic nephroureterectomy (HARN) was performed via a Gibson incision at our institution. Based on accumulated experiences and skills, unilateral extraperitoneal nephroureterectomy could be practiced through a paramedian incision [13]. During this period, a camera port was required to insert a laparoscope and ensure the security of* en bloc* ligation of renal vessels; an extended paramedian incision could immediately be made as if major bleeding needed to be treated. After becoming progressively proficient in surgical anatomy of the retroperitoneal space, better exposure could be attained and the renal hilum might be directly visualized without the need of laparoscopy. Additionally, palpitation of the renal pedicle, digital dissection, and retraction were utilized to collaborate with perirenal dissection. In consideration of postoperative analgesia and functional recovery, an infraumbilical midline incision was attempted to accomplish unilateral extraperitoneal nephroureterectomy, which had been done by one single surgeon in more than 200 patients.

For the purpose of good exposure of the retroperitoneum, two important surgical steps are addressed. First, division of the round ligament or spermatic cord facilitates mobilization of the peritoneum and its contents [14, 15]. Anatomically, there are two cord-like structures passing forwards in the peritoneal fold and entering the inguinal canal by the internal ring, considered to be the round ligament or spermatic cord in the female and the male, respectively. Mass ligation of these structures can separate the peritoneum off the iliac vessels and the psoas muscle is exposed laterally and posteriorly to help in identifying and skeletonizing the ureter cephalad. Second, along with a Bookwalter retractor system, two customized long right-angle retractors, of which the blade is 15 or 23 cm, are used to push the peritoneum medially and anteriorly. With traction on the peritoneum, the intraperitoneal contents may be naturally retracted and protected, lowering the risk of adjacent organ interference or injury, even in the peritoneal dialysis patient with a history of sclerosing peritonitis; a wide space may be provided for ureterolysis, perirenal dissection, and isolation of the renal pedicle.

On the other hand, in order to complete laparoscopic bilateral upper urinary tract surgery in single session, utilizing gravity to maneuver the bowel is of great concern. Some experts alternated inflatable air tourniquet cuffs or gel rolls on each side of the patient's back, or tilted the table to facilitate displacing the bowel by gravity [4, 9, 16, 17], thus saving the time of repositioning and redraping. All patients in our series were operated in the modified dorsal lithotomy position, with both arms outstretched, throughout the whole procedure. Plenty of time could be preserved, attributing to no change of patient's position. This could explain why our operative time was significantly shorter compared to those of published studies [7–9]. By virtue of minimally invasive surgical techniques with laparoscopic instruments, as well as tactile feedback and direct three-dimensional visualization, the method presented here permitted a faster upper urinary tract extirpation.

As for shorter interval to oral intake, it might be ascribed to shorter operative duration; other perioperative parameters appeared to be similar. Owing to very low premiums of the National Health Insurance in Taiwan, patients usually would not be discharged until being fully recovered. That is why our hospital stay could not be shortened. Comparing other studies with regard to synchronous bilateral renal surgery [3–6], there was significantly more blood loss in our series. The amount of bleeding might be correlated with the extent of surgery. Radical hysterectomy and bilateral salpingo-oophorectomy were simultaneously performed, partly explaining why the blood loss was significantly higher than that of other smaller-scale surgeries. Nevertheless, our surgical time and postoperative convalescence were still comparable.

With regard to oncologic control, 10 patients remained cancer-free at a follow-up of at least 19 months, the longest being 105 months. This might be attributed to the broadened indication for CUTE at our institute, including primary bladder cancer in uremic patients.

Preoperatively undetectable malignancy, such as small RCC or superficial UC, in the upper tract could be* en bloc* removed in single session. Six patients were postoperatively confirmed as having concurrent UC or ccRCC in our series, even after carefully reviewing preoperative CT scans. Although no other study is available to substantiate this finding, it is confirmed that an aggressive surgical approach should be executed to treat UC in uremic patients regarding its notorious behavior. However, there are still contraindications to our techniques, including advanced upper tract tumors and lymph node metastasis identified preoperatively, due to difficulty in attaining negative surgical margins and removing enough lymph nodes. In another aspect, it should be concerned that the risk of ruptured collecting system exists, especially in patients with moderate or severe hydronephrosis. There was no aforementioned complication encountered in our patients because most of them had localized upper tract tumors in atrophic kidneys.

The present study was limited by its retrospective nature and small case number. The amount of analgesia and quality of life were not evaluated as well postoperatively. Despite good exposure of the retroperitoneal space, it is still a confined space to perform perirenal dissection, even in hand-assisted retroperitoneoscopic surgery. Without proficient anatomical knowledge of the retroperitoneal space and sophisticated minimally invasive surgical skills, peritoneal violation might be encountered, thereby causing the bowel to protrude through the peritoneal defect and hinder access to the surgical field. Reproducibility may be an issue, but, in experienced hands, it can be done efficiently and safely with minimal morbidity. There were still two cases of postoperatively prolonged ileus, and it might be related to the transperitoneal approach for radical cystectomy. A prospective randomized study with a larger sample and long-term follow-up would be needed to demonstrate the possible benefit more conclusively.

The concept of peritoneal mobilization promotes our surgical evolution, leading to better exposure of the retroperitoneum. Even though peritoneal violation is present, the customized long right-angle retractors and moist laparotomy pads may be utilized to overcome this obstacle. Our modified technique provides an expanded indication for CUTE. It is recommended that this method be implemented in dialysis patients with multifocal UC in which upper tract tumors are localized without lymph node metastasis or those with primary bladder cancer eligible for radical cystectomy, thus eliminating the preoperatively undetectable tumors and reducing the likelihood of metachronous malignancy. Gynecologic organs can simultaneously be removed to achieve better oncological control. Without pneumoperitoneum and repositioning, it can be performed via an infraumbilical midline mini-incision, and the entire specimen can be extracted* en bloc* with intact urothelial continuity.

## 5. Conclusions

This modified single mini-incision CUTE provides a time-saving method for dialysis patients with UC, eliminating preoperatively undetectable malignancy and reducing metachronous recurrences; perioperative complications associated with pneumoperitoneum and repositioning can be averted as well. Good cancer control and early convalescence may both be achieved in experienced hands.

## Figures and Tables

**Table 1:** Patient demographics and perioperative parameters.

Pt	Age (y)	Duration of HD (yr)	BMI (kg/m^2^)	ECOG	Clinically surgical indication	Hydronephrosis	Pathologic tumor location (T stage)^a^	Comorbidity
1	63	9	21.4	0	UB & LU	Left	UB(is) & LU(1) **LK ccRCC(1a)**	HTN
2	50	14	21.7	0	UB & RU	Right	UB(1) & RU(3)	HTN
3	61	8	25.9	1	UB & RU∗∗	Right	UB(a) & RU(a)	HTN, HCC, cirrhosis, hepatitis B & C, SHPT
4	75	6	31.5	0	UB & LU LK	Left	UB(a) & LU(a) LK(a)	None
5	63	17	17.1	1	UB & LK	Bilateral	UB(1) & LK(3) **LU(a)**	HTN, hepatitis C, pulmonary TB
6	52	4	31.8	0	UB & RK	No	UB(1) & RK(1)	HTN, DM
7	67	1	20.4	1	BK & RU	Right	BK(a) & RU(1)	HTN, CHF
8	80	6	17.0	1	UB & LU∗∗	Bilateral	UB(2a) & LU(a) **RK(a) & RU(a)**	HTN, DM, moderate MR
9	58	9	21.3	1	UB & RU	No	UB(is) & RU(1) **LU(is)**	SHPT
10	70	2	22.6	1	UB & RU∗∗	Right	UB(1) & RU(1)	HTN, old CVA, parkinsonism
11	57	8	22.2	1	UB∗∗	Left	UB(2a) **LU(is) & RU(3)**	SHPT
12	57	13	17.3	1	UB∗∗	Left	UB(1)	Pulmonary TB
13	53	10	21.5	0	UB	No	UB(1) **LU(a)**	HTN, hepatitis B
14	48	11∗	21.2	1	UB	Left	UB(1)	HTN, hepatitis C, SHPT, peritonitis∗∗∗

HD, hemodialysis; BMI, body mass index; ECOG, eastern cooperative oncology group performance status; UB, urinary bladder; LU, left ureter; RU, right ureter; LK, left kidney; RK, right kidney; BK, bilateral kidney; ccRCC, clear cell renal cell carcinoma; HTN, hypertension; HCC, hepatocellular carcinoma; SHPT, secondary hyperparathyroidism; TB, tuberculosis; DM, diabetes mellitus; CHF, congestive heart failure; CVA, cardiovascular accident.

^a^All patients had high-grade urothelial carcinoma.

∗Peritoneal dialysis.

∗∗Surgical indication was recurrent urothelial cancer.

∗∗∗Continuous ambulatory peritoneal dialysis (CAPD) related sclerosing peritonitis.

**Table 2:** Comparison of outcomes from concurrent upper and lower urinary tract surgery.

Author	Number	Extent of surgery	Age (y)	Operative time (min)^a^	Blood loss (mL)^a^	Hospital stay (d)^a^	Time to intake (hr)^a^	Complication (%)^b^
El-Galley et al., 2011 [3]	36	BN	N/A	222	175∗∗∗	3.0∗∗∗	N/A	22.2
Chueh et al., 2002 [4]	7	BNU	51.6	294∗∗	218∗∗	8.8∗	39.0	14.3
Tai et al., 2009 [5]	33	BNU	52.4	309∗∗	226∗∗	10.2	58.0	12.1
Ou and Yang, 2011 [6]	13	BNU	60.0	215	216∗∗	13.8	60.0	7.7
Ou and Yang, 2011 [7]	10	CUTE	57.6	328∗∗∗	628	14.7∗	62.4∗	10.0
Li et al., 2009 [8]	5	CUTE	58.0	492∗∗∗	378	12.2	72.0∗∗	80.0
Lin et al., 2011 [9]	5	CUTE	66.6	397∗∗∗	532	10.8	91.2∗∗∗	20.0
Present study	14	CUTE	61.0	237.5	560.7	12.1	48.0	21.4

BN, bilateral nephrectomy; BNU, bilateral nephroureterectomy; CUTE, complete urinary tract exenteration.

^a^One sample *t*-test.

^b^Chi-square test.

*P < 0.005, ∗∗*P* < 0.01, ∗∗∗*P* < 0.001 for compared to present study.

N/A, not available.
